# Supplementary material for: Impact of the “Looking after my health after cancer” peer-led active patient education program on cancer survivors and their caregivers: A qualitative study
Source: PLoS One. 2023 Feb 24;18(2):e0282018. doi: 10.1371/journal.pone.0282018 (PMC9955581; doi:10.1371/journal.pone.0282018)
Supplement: Attachment — Submitted filename: PONE-D-22-19858.docx [file pone.0282018.s001.docx]

**PONE-D-22-19858 comments**

The authors conducted research that touches on a very important topic. They employed a phenomenology approach to the design.

The objectives of the study are stated as exploring: how cancer survivors and their caregivers rate the experience of participating in the program, the extent to which the program helps them understand and address their unmet felt needs, and the extent to which it helps them improve their activation for self-care and self-management.

Methods

Overall, the methods section is well outlined with details. I would however have liked to see the methods presented using the five steps/stages of the phenomenology design used, as outlined below:

**1.** Bracketing and phenomenological reduction.

**2.** Delineating units of meaning.

**3.** Clustering of units of meaning to form themes.

**4.** Summarising each interview, validating it and where necessary modifying it.

**5.** Extracting general and unique themes from all the interviews and making a composite summary.

Line 178 ‘has’ should be ‘had’

Results.

Table 2

It is not obvious what the letter 'M' in the second column represents. Is it mean or median?

Secondly, this value should be a measure of central tendency and its appropriate measure of dispersion in parentheses e.g., mean (sd), median (iqr)

The ratio of Females to Males is quite large. Was this part of the design or a flaw in sampling? Was it not necessary to have equal gender representation? This is especially important because you mention that you aimed at achieving a heterogeneous sample.

In my opinion, it would have been better to see the results presented per objective. The way they have been presented leaves one wondering if there was a reflection on the individual objectives of the study in a way that would enable the reader to understand if the study questions were answered or not. I would suggest that the results are reorganised per the objectives.

Line 255. “Satisfaction with the program and the positive learning experience.” Could these be two independent themes? There are several areas where the results are presented this way. I would imagine that the main themes would be meaningful enough to stand on their own. Either the wrong title of themes was used, or more than two or more themes are being combined under one heading in a way that makes them seem different.

Line 416, 417. “Having a more positive view of their experience and becoming active in self-care and more empowered in self-management of their condition.” Are these not two independent themes? Being more empowered in self-management (self-efficacy) is different from having a positive view (self-image)

There are numerous areas in the results section where only positive findings were reported. I might have missed this, but it is rare for any study of this kind not to have a single negative or surprising or unexpected finding. I am not doubting the results as presented. I am simply wondering if the authors took time to either ask about things that didn’t go well or any findings that might have not been positive. These too are important to report about, if they exist.

**Discussion**

When researchers use comparative methods, they organise groups of findings with the objective of developing new theories about phenomena. It is not obvious what new theory about interventions of this kind was discerned from the process. What is the overriding communication finding(s) from the study? This is not well articulated.

Line 458-459 “The success of this program is attributed to the fact that it is peer led, that is, mediated by people who have experienced a similar situation and have received training to become leaders and run the sessions.”

For most interventions of this kind, their effects cannot be attributed to one factor. Moreover, the study was not designed to show cause and effect. it is therefore not obvious that the fact that it was per led is the only reason it was successful. This was not a process evaluation study. I am, therefore, not sure you had all the variables to explain why the intervention worked the way it did. At best, the authors can only hypothesize about the factors that could have contributed to its success.

Strengths and limitations section:

One potential limitation is the use of one method of data collection – focus groups. Studies of this kind could benefit from individual interviews and focus groups unless the goal is to achieve consensus, which was not the case in this study.

Line 506: triangulation

Triangulation is often used in dealing with different groups and different research methods. It is not clear how triangulation was done in this study. It would be great if the authors described this in detail.

Reflexivity

It is good practice to reflect on the authors’ own biases in designing and implementing qualitative studies. For this reason, authors of qualitative studies, especially interpretive designs such as the one used in this study are advised to reflect on how their experiences might have influenced the conduct of the study. The authors are therefore advised to include a note on reflexivity. How did the researchers' training and life and professional experiences influence the study design, and data collection, interpretation and reporting?
