# Supplementary material for: Impact of the “Looking after my health after cancer” peer-led active patient education program on cancer survivors and their caregivers: A qualitative study
Source: PLoS One. 2023 Feb 24;18(2):e0282018. doi: 10.1371/journal.pone.0282018 (PMC9955581; doi:10.1371/journal.pone.0282018)
Supplement: Attachment — Submitted filename: Response to Reviewers PONE-D-22-19858R1.docx [file pone.0282018.s004.docx]

January 30^th^, 2023.

**Editor-in-Chief**, ***PLOS One***

Dear Dr Chenette,

Manuscript PONE-D-22-19858R1, entitled ‘*Impact of the “Looking after my health after cancer”* *peer-led active patient education program on cancer survivors and their caregivers: A qualitative study*’

We sincerely appreciate the interest of the *PLOS One* journal in our work, and we are grateful for the comments provided. These comments have undoubtedly helped us to improve our manuscript.

Please find enclosed a revised version of the manuscript (PONE-D-22-19858R1) in which we have addressed all the comments of the Academic Editor and implemented her suggestions. All changes are shown using the Track changes tool and are also highlighted in yellow. All co-authors have approved these changes.

Thank you again for the privilege of having our work reviewed, and we hope that you find our responses satisfactory.

We look forward to your final decision regarding our manuscript.

Our very best,

The authors.

**LIST OF RESPONSES TO THE ACADEMIC EDITOR**

| **Academic Editor Comments**  **Sandra Boatemaa Kushitor, Ph.D.** | **Authors’ Revisions** |
| --- | --- |
| Kindly edit your manuscript and remove all first person words. Avoid I and we, use phrases such as 'the researcher', the authors ectera. | We have kindly re-edited the manuscript, removing all the first-person words. We have avoided words like “we”, “our”, “us”.  We are grateful for the privilege of having our manuscript reviewed again and for the valuable suggestions which we believe have helped us to improve it. |
